# Kallikrein-Related Peptidase 6 Is Associated with the Tumour Microenvironment of Pancreatic Ductal Adenocarcinoma

**DOI:** 10.3390/cancers13163969

**Published:** 2021-08-05

**Authors:** Juliana B. Candido, Oscar Maiques, Melanie Boxberg, Verena Kast, Eleonora Peerani, Elena Tomás-Bort, Wilko Weichert, Amiram Sananes, Niv Papo, Viktor Magdolen, Victoria Sanz-Moreno, Daniela Loessner

**Affiliations:** 1 Centre for Tumour Microenvironment, Barts Cancer Institute, Queen Mary University of London, London EC1M 6BQ, UK; j.candido@qmul.ac.uk (J.B.C.); o.m.carlos@qmul.ac.uk (O.M.); e.f.peerani@qmul.ac.uk (E.P.); e.tomasbort@qmul.ac.uk (E.T.-B.); v.sanz-moreno@qmul.ac.uk (V.S.-M.); 2 Institute of Pathology, Technical University of Munich, 81657 Munich, Germany; Melanie.boxberg@tum.de (M.B.); wilko.weichert@tum.de (W.W.); 3 Max Bergmann Center of Biomaterials Dresden, Leibniz Institute of Polymer Research Dresden e.V., Hohe Straβe 6, 01069 Dresden, Germany; Kast@ipfdd.de; 4 Avram and Stella Goldstein-Goren Department of Biotechnology Engineering and The National Institute of Biotechnology in the Negev, Ben-Gurion University of the Negev, Beer-Sheva 8410501, Israel; amiramsa@post.bgu.ac.il (A.S.); papo@bgu.ac.il (N.P.); 5 Department of Obstetrics and Gynaecology, Technical University of Munich, 81675 Munich, Germany; viktor.magdolen@tum.de; 6 Department of Chemical Engineering and Department of Materials Science and Engineering, Faculty of Engineering, Monash University, Melbourne, VIC 3800, Australia; 7 Department of Anatomy and Developmental Biology, Biomedicine Discovery Institute, Faculty of Medicine, Nursing and Health Sciences, Monash University, Melbourne, VIC 3800, Australia

**Keywords:** pancreatic cancer, kallikrein-related peptidase 6, tumour microenvironment, tumour spheroids

## Abstract

**Simple Summary:**

Kallikrein-related peptidases have tumour-biological roles and are dysregulated in many cancers. Only a few studies have reported their upregulation in pancreatic cancer and linked them to poor prognosis. By interrogating publicly available and our own datasets, we studied their expression in patient-derived tissues and pancreatic cancer cells. We found several kallikrein-related peptidases that were upregulated, in particular kallikrein-related peptidase 6 at the forefront of the tumour area. We then tested the effect of a kallikrein-related peptidase 6 inhibitor on cancer cell functions. Because the majority of patients present with inoperable disease, a targeted therapeutic intervention may have a positive impact on the survival of this patient population.

**Abstract:**

As cancer-associated factors, kallikrein-related peptidases (KLKs) are components of the tumour microenvironment, which represents a rich substrate repertoire, and considered attractive targets for the development of novel treatments. Standard-of-care therapy of pancreatic cancer shows unsatisfactory results, indicating the need for alternative therapeutic approaches. We aimed to investigate the expression of KLKs in pancreatic cancer and to inhibit the function of KLK6 in pancreatic cancer cells. KLK6, KLK7, KLK8, KLK10 and KLK11 were coexpressed and upregulated in tissues from pancreatic cancer patients compared to normal pancreas. Their high expression levels correlated with each other and were linked to shorter survival compared to low KLK levels. We then validated KLK6 mRNA and protein expression in patient-derived tissues and pancreatic cancer cells. Coexpression of KLK6 with KRT19, αSMA or CD68 was independent of tumour stage, while KLK6 was coexpressed with KRT19 and CD68 in the invasive tumour area. High KLK6 levels in tumour and CD68+ cells were linked to shorter survival. KLK6 inhibition reduced KLK6 mRNA expression, cell metabolic activity and KLK6 secretion and increased the secretion of other serine and aspartic lysosomal proteases. The association of high KLK levels and poor prognosis suggests that inhibiting KLKs may be a therapeutic strategy for precision medicine.

## 1. Introduction

Pancreatic tumours are cancers of substantial unmet needs, with less than 10% of patients surviving 5 years after diagnosis [1]. By 2030, they are predicted to be the second leading cause of cancer-related deaths [2]. Pancreatic ductal adenocarcinoma (PDAC) is the most common subtype of pancreatic cancer [3]. The efficacy of therapeutics and the treatment of PDAC is hampered by the fibrotic tumour stroma, which is made up of a dense and crosslinked extracellular matrix (ECM), and the heterogeneity within tumours and between patients [2]. The standard-of-care treatment of advanced disease is a combination of gemcitabine and nab-paclitaxel, which prolongs patient survival by only a few months. Metastatic disease burden is a primary cause of death in patients with PDAC; hence, treatments aimed at preventing the dissemination of tumour cells may be an alternative therapeutic approach. Treatment responses and disease progression are mediated by the tumour microenvironment (TME), which is a complex and dynamic niche that interlinks various cell populations, the ECM, secreted factors and signalling molecules [4].

As cancer-associated factors, kallikrein-related peptidases (KLKs) are components of the TME and considered attractive targets for the development of novel treatments. KLKs are secreted trypsin and chymotrypsin-like serine proteinases that degrade a variety of molecules, including ECM proteins, cytokines and growth factors. They are the largest contiguous cluster of serine proteases in the human genome. Tissue kallikrein (KLK1) was first identified in the pancreas, and the name of this group of proteases is derived from the Greek word for pancreas—‘kallikreas’. There are 15 family members that have roles in physiology, such as regulation of skin renewal, semen liquefaction, tooth development and in the cardiovascular system, as well as pathophysiology [5]. In many cancer types, abnormal KLK levels are linked to tumour cell proliferation, migration and invasion [6].

In pancreatic cancer, KLKs may have regulatory functions in the TME, as this niche represents a rich substrate repertoire. Only a few studies reported that KLKs are upregulated in pancreatic cancer and linked aberrant KLK levels to poor prognosis. High KLK6, KLK7 and KLK10 gene and protein levels were associated with shorter survival of PDAC patients [7,8,9]. KLK6 and KLK10 were highly upregulated in PDAC compared to normal and benign tissues [10]. KLK7 was also highly expressed in PDAC but not in normal pancreas tissues [11]. KLK6, KLK7, KLK8 and KLK10 were detected in supernatants from human PDAC cells using KLK-specific ELISAs [12]. Biochemical studies using recombinant proforms of KLKs and their proteolytic active forms and other proteases showed that KLK6 is the starting protease of the proteolytic network in PDAC and can be activated by plasmin and urokinase plasminogen activator (uPA) [13,14]. KLK6 was originally discovered as a myelencephalon-specific trypsin-like serine proteinase present in the central nervous system [15]. It was found highly expressed by inflammatory or immune cells, including macrophages, in neurological disorders, such as Alzheimer’s and Parkinson’s disease and multiple sclerosis [16]. KLK6 knockout mice showed a delayed onset and less severe symptoms of multiple sclerosis and reduced inflammatory cells and cytokine levels [17].

In this study, we aimed to investigate the biochemically proposed KLK network in PDAC using patient-derived samples and the tumour-biological role of KLK6 in human PDAC cells. Therefore, we interrogated publicly available PDAC datasets and performed expression analyses using our own patient cohorts to identify the PDAC-specific KLK cluster and the association with patient prognosis. We then correlated KLK6 expression with tumour, stroma and immune cell areas. Lastly, we used KLK6-expressing human PDAC cells to test a proteolysis-resistant KLK6 inhibitor and its effects on KLK6 expression; secretion; cell functions, for example cell proliferation and migration; and KLK6-interacting factors using a preclinical tumour spheroid model. The APPI-4M KLK6 inhibitor is proteolytic stable when incubated with recombinant proteolytic active KLK6 over several days and is a human amyloid precursor protein inhibitor that contains the Kunitz-type protease inhibitor domain with high binding affinity to KLK6 [18].

## 2. Materials and Methods

### 2.1. Cell Culture

Human pancreatic cancer (AsPC1, BxPC3, Capan2, MiaPaCa2 and Panc1) and colon cancer cells (HT29 and SW480) were purchased from ATCC and grown in DMEM or RPMI supplemented with 10% foetal calf serum (FCS) and 1% penicillin/streptomycin (P/S). Human pancreatic cancer Colo357 cells were kindly provided by Dr Stéphanie Kermorgant, Barts Cancer Institute. Human ovarian cancer OV-RSV and OV-KLK6 cells were previously established [19]. Cells were regularly tested for mycoplasma and grown to 70–80% confluency before use.

### 2.2. 3D Cell Culture and Analyses

Cells were grown encapsulated in polyethylene glycol (PEG)-based hydrogels (proteolytic degradable; without RGD motif or RGD-functionalised; PC17; QGel, QGel SA, Lausanne, Switzerland) at a cell density of 3.5 × 10^5^ cells/mL over 14 days, with 5 replicates per hydrogel composition. Briefly, the cell-containing hydrogel precursor solution was sandwiched between two sterile, Sigmacote^®^ pre-treated, hydrophobic glass slides and 1.5 mm spacers. After polymerisation at 37 °C/5% CO_2_ for 35 min, hydrogel discs were removed from the glass slides and immersed in culture medium in 48-well plates. Metabolic activity and cell proliferation were assessed performing AlamarBlue and CyQuant assays (DAL1025 and C7026, Thermo Fisher Scientific, Loughborough, UK) as described and represented as fluorescence intensity and DNA concentration, respectively [19]. The mechanical properties of cell-containing hydrogels were determined by unconfined compression testing using an Instron 3342 machine equipped with a 10 N load cell. Young’s modulus was determined by calculating the slope at the linear region of the stress–strain graph. To determine the effect of the APPI-4M KLK6 inhibitor [18], 3D cell cultures were treated on Day 7 with 100 nM for 7 days, with treatment renewals every other day. The secretion of proteases in conditioned medium from the 3D cell cultures was assayed using a commercially available proteome profiler human protease array kit (ARY021B, R&D Systems, Abingdon, UK). Profiles of mean spot pixel density were created using a transmission-mode scanner and image analysis software.

### 2.3. Gene Expression

RNA was isolated either using the TRIzol reagent (Sigma-Aldrich, Dorset, UK) or with the RNeasy Microkit (74004, Qiagen, Manchester, UK) according to the manufacturer’s instructions with additional on-column DNase digestion. Hydrogel samples were disrupted in gentleMACS M-tubes (130-093-236, Miltenyi Biotec, Surrey, UK) placed in a gentleMACS dissociator. Up to 1 μg of RNA was reverse transcribed using the high-capacity cDNA Reverse Transcription kit (4368814, Thermo Fisher Scientific). A volume of 10 μL cDNA was combined with 10 μL of Universal TaqMan PCR Mastermix (4304437, Thermo Fisher Scientific) and 1 μL of the primer probe KLK6 Hs00160519_m1 and HPRT1 Hs02800695_m1 (Thermo Fisher Scientific). Samples were run in duplicates using a StepOnePlus real-time PCR machine (Applied Biosystems, Thermo Fisher Scientific). ΔCt values were calculated by subtracting the Ct value of the gene of interest from the Ct value of the housekeeping gene.

### 2.4. Protein Expression

Protein was extracted by lysing cells in RIPA buffer (R0278, Sigma-Aldrich) containing 1:10 complete mini-EDTA protease inhibitor (11836153001, Roche, Welwyn Garden City, UK) and 1:100 phosphatase inhibitors (P5726 and P0044, Sigma-Aldrich) targeting tyrosine protein phosphatases, acid and alkaline phosphatases, as well as serine-threonine protein phosphatases. Protein concentrations were determined using a BCA assay. Then, 33 μg of sample was loaded on 4–12% NuPAGE Bis-Tris gels (NP0321BOX, Thermo Fisher Scientific) and run in MOPS SDS Running Buffer (NP001 Thermo Fisher Scientific) with NuPAGE LDS Sample Buffer (NP007, Thermo Fisher Scientific) and NuPAGE Sample Reducing Agent (NP004, Thermo Fisher Scientific) and transferred onto a PVDF membrane (NEF1002001PK, Perkin Elmer, Waltham, MA, USA) in NuPAGE transfer buffer (NP00061, Thermo Fisher Scientific). Membranes were blocked with 5% skimmed milk powder (Marvel) in TBS containing 0.1% Tween20 for 1 h at room temperature. Primary antibodies (KLK6#427 1:500, rabbit; αTubulin 1:1000, 2125 (11H10), Cell Signaling Technology, Danvers, MA, USA) were diluted in blocking buffer and incubated overnight at 4 °C. Then, membranes were incubated with HRP-conjugated antibodies (anti-rabbit 1:2000, 7074, Cell Signaling) for 1 h at room temperature. HRP activity was visualised with Amersham ECL (RPN2232, GE Healthcare, Chalfont St Giles, UK) on an Amersham Imager 600 (GE Healthcare).

### 2.5. Functional Assays

Analysis of cell proliferation and migration was carried out using the Incucyte ZOOM live cell analysis system. Prior to cell seeding, 96-well plates were coated with collagen type-I (10 µg/mL, Gibco, Thermo Fisher Scientific) or poly-L-lysine (10 µg/mL, Sigma-Aldrich) overnight at 4 °C and cells were serum-starved in medium containing 1% FCS and 1% P/S. Then, cells were seeded (proliferation: 1 × 10^4^ cells/well; migration: 8 × 10^4^ cells/well, Capan-2 at 10 × 10^4^ cells/well) in complete growth medium ± APPI-4M KLK6 inhibitor (final concentration of 100 nM/well) overnight at 37 °C and thereafter imaged for 96 h (proliferation) or 48 h (migration; scratches generated prior to imaging start), with 5 wells per group.

### 2.6. In Situ Hybridisation

A new tissue microarray (TMA) was established using formalin-fixed, paraffin-embedded (FFPE) biopsies from individuals (*n* = 41) diagnosed with primary PDAC (Table S1). Biopsies were examined by a pathologist (O.M.) to represent each specimen by two cores (1 mm diameter) from the tumour body and two cores from the invasive tumour areas. Peritumoral cores were included for specimens that had healthy tumour-adjacent pancreas tissue. Grading and staging followed the most recent World Health Organization criteria. Samples were processed by the IRBLleida (PT17/0015/0027) and HUB-ICO-IDIBELL (PT17/0015/0024) Biobanks integrated in the Spanish National Biobank Network and Xarxa de Bancs de Tumours de Catalunya following standard operating procedures with the respective approval of the local Ethics Committee. Freshly cut tissue sections were stained using the KLK6 probe (RNAscope probe Hs-KLK6, 522111) mixed 1:50 with one of the following probes: Hs-KRT19-C2 (426221-C2), Hs-ACTA2-O1-C2 (444771-C2) or Hs-CD68-C2 (560591-C2) using the RNAscope^®^ 2.5 HD Duplex Reagent Kit (322430). Slides were imaged using the NanoZoomer S210 slide scanner (Hamamatsu, Welwyn Garden City, UK). Staining quantification was performed with the QuPath 0.1.2 software following the recommended protocol [20]. Scans were loaded into QuPath, and the image type was changed to ‘brightfield other’. Channel 2 was set up to detect the green staining (KLK6) and channel 3 to detect the red staining (KRT19, αSMA or CD68). The TMA dearrayer was used to identify and label individual cores. Next, cell segmentation was performed using ‘cell detection’. Chromogen detection and quantification was performed using ‘subcellular detection’, and thresholds were set according to the staining intensity. Lastly, a ‘detection classifier’ using the trees classification algorithm combined with the intensity information was created to classify the tumour, stroma, immune cell and normal tissue areas. Finally, each core was scored using the H-score method for each chromogen based on the staining intensity (0/no staining, 1/weakly positive, 2/moderately positive, 3/strongly positive) and percentages of stained area.

### 2.7. Immunohistochemistry

A previously established TMA with primary resected specimens from individuals (*n* = 262) that received partial pancreatoduodenectomy for PDAC between 1991 and 2006 at the Charité University Hospital (Berlin, Germany) was used (Ethical Approval Number: EA1/06/2004) [21]. Grading and staging followed the World Health Organization recommendations at the time of cohort generation. Immunohistochemical analysis was performed on 2 µm FFPE tissue sections with three tumour cores per specimen. An automated immunostainer (Bond RXm system, Leica Biosystems, Milton Keynes, UK) with an ultraView Universal DAB detection kit (Ventana Medical Systems, Roche) was used for staining with the KLK6#427 (1:75, rabbit) antibody. Appropriate positive and negative controls were run in parallel. Sections were examined by a pathologist (M.B.) to assess the staining intensity of the tumour, stroma and immune cell areas, followed by quantification via QuPath 0.1.2 as above.

### 2.8. TCGA and APGI Analysis

The TCGA PDAC dataset [22] was downloaded from FIREHOSE (Broad Institute, Available obline: https://gdac.broadinstitute.org/, accessed on 27 March 2018) to perform differential expression and statistical analyses using the R programming language. The normalised readout was log2 transformed for graphical presentation. The APGI PDAC dataset [23] was analysed using the online tool cBioPortal to perform differential expression and statistical analyses. The normalised readout was log2 transformed for graphical presentation.

### 2.9. Statistical Analysis

Data are presented as graphs showing mean ± standard error of the mean or as a minimum-to-maximum boxplot. Numeric data were analysed using two-tailed unpaired Student’s *t*-tests if data followed a normal distribution and using Mann–Whitney U-tests if data did not follow a normal distribution. Wilcoxon tests, one-way ANOVA with Dunnett or Tukey post hoc tests and two-way ANOVA were also carried out. Statistically significant differences are indicated (* *p* ≤ 0.05; ** *p* ≤ 0.01; *** *p* ≤ 0.001). Kaplan–Meier survival curves were generated with GraphPad Prism8 (GraphPad Software Inc, La Jolla, CA, USA) using the log-rank (Mantel–Cox) test.

## 3. Results

### 3.1. KLK Coexpression in PDAC Tissues

**Figure 1:**
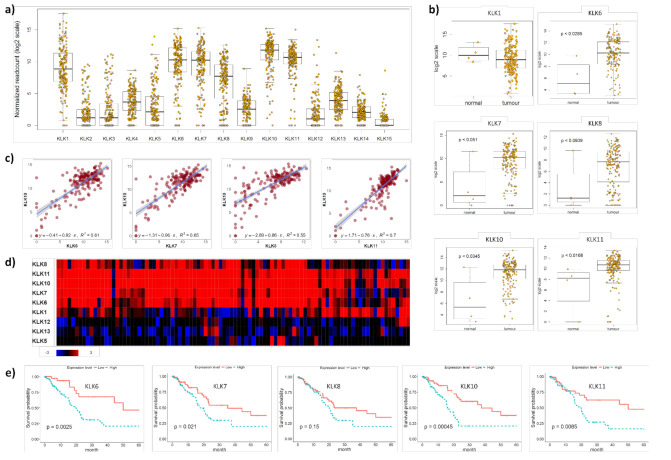
KLK co-expression in PDAC tissues. (**a**) On RNA sequencing, KLK1, KLK6, KLK7, KLK8, KLK10 and KLK11 were identified as highly upregulated in PDAC tissues. Yellow dots represent individual PDAC patients. (**b**) KLK6, KLK7, KLK8, KLK10 and KLK11 were significantly upregulated in PDAC compared to normal pancreas tissues (*p* ≤ 0.05), with KLK8 showing a trend (*p* = 0.0939). Yellow dots represent individuals. (**c**) Pearson correlation coefficients indicating a significant correlation between KLK10 and KLK6 (R^2^ = 0.61), KLK7 (R^2^ = 0.65), KLK8 (R^2^ = 0.55) and KLK11 (R^2^ = 0.7) levels (*p* ≤ 0.05). Purple dots represent individual patients. (**d**) On whole-genome sequencing, KLK6, KLK7, KLK8, KLK10 and KLK11 were identified as a highly upregulated gene cluster in PDAC compared to normal pancreas tissues. Rectangles represent individual patients; red—upregulation; blue—downregulation; black—not differentially expressed. (**e**) Kaplan–Meier analysis indicated that high KLK6 (*p* = 0.0025), KLK7 (*p* = 0.021), KLK10 (*p* = 0.00045) and KLK11 (*p* = 0.0085) expression levels were significantly associated with a shorter 5-year survival than low expression levels.

The integrated genomic characterisation of patients diagnosed with PDAC (*n* = 178) was carried out through the TCGA research network [22]. Interrogating this dataset, we found that KLK1, KLK6, KLK7, KLK8, KLK10 and KLK11 were highly upregulated in PDAC tissues (Figure 1a).

We found that KLK6, KLK7, KLK8, KLK10 and KLK11 were significantly upregulated in PDAC compared to normal pancreas tissues, while KLK1 levels did not differ between PDAC and normal pancreas tissues (Figure 1b). To identify a correlation between different members of the PDAC-specific cluster, we plotted the mRNA levels of KLK10, the highest expressed kallikrein in the TCGA PDAC dataset [22], against the other members in this cluster and found a significant correlation with all of them (Figure 1c and S1a). Interrogating a larger PDAC patient cohort (*n* = 456) from the APGI [23], we confirmed the expression of this PDAC-specific KLK cluster. KLK1, KLK6, KLK7, KLK8, KLK10 and KLK11 were highly upregulated in PDAC compared to normal pancreas tissues. In 21% of the cases, KLK6, KLK7, KLK8, KLK10 and KLK11 were coexpressed, while KLK1, KLK5, KLK12 and KLK13 were not coexpressed and downregulated (Figure 1d), which was also reported earlier for KLK12 and KLK13 [7]. In line with the two published reports on the association of high KLK6, KLK7 and KLK10 expression levels with shorter survival of PDAC patients [7,8], we also found that high mRNA levels of KLK6, KLK7, KLK10 and KLK11 were linked to shorter survival compared to low mRNA levels (Figure 1e). Using the KLK-CANMAP, a tool that includes several other cancer datasets, we again found the KLK6, KLK7, KLK8 and KLK10 cluster, with the exception of KLK11, which was upregulated in PDAC compared to normal pancreas tissues (Figure S2a). High mRNA levels of KLK6, KLK7, KLK8, KLK10 and KLK11 were linked to shorter survival compared to low mRNA levels (Figure S2b). Overall, the mRNA expression data indicate that KLK6, KLK7, KLK8, KLK10 and KLK11 are highly upregulated and coexpressed in PDAC and linked to shorter survival.

### 3.2. KLK6 Expression in PDAC Tissues

**Figure 2:**
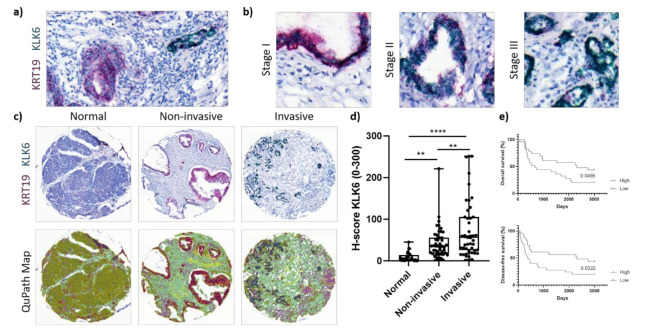
KLK6 mRNA expression in PDAC tissues. (**a**) Representative image of dual in situ hybridisation of KLK6 (green) and KRT19 (red) in PDAC tissues, indicative of a high copy number of both mRNAs. (**b**) Representative images of dual in situ hybridisation of KLK6 (green) and KRT19 (red) depict high copy numbers of KLK6 independent of tumour stage. (**c**) Representative images of dual in situ hybridisation of KLK6 (green) and KRT19 (red) in normal pancreas and PDAC tissues and the respective QuPath map images below. (**d**) KLK6 mRNA was upregulated in PDAC compared to normal pancreas tissues, with invasive tumour areas having higher mRNA levels compared to the noninvasive counterparts. (**e**) Kaplan–Meier analysis indicated that high KLK6 expression levels were significantly associated with shorter overall (*p* = 0.0485) and disease-free (*p* = 0.0322) survival than low expression levels (H-score cut-off 55). Asterisks indicate statistical significance (** *p* ≤ 0.01 and **** *p* ≤ 0.001).

Carrying out our own mRNA expression analysis, we performed dual in situ hybridisation using our own PDAC TMA and a previously established PDAC TMA. Biochemical studies using recombinant proforms of KLKs and their proteolytic active forms and other proteases showed that KLK6 is the starting protease of the proteolytic network in the PDAC-specific cluster and can be activated by plasmin and uPA [13,14]. Thus, we sought to determine the mRNA expression of KLK6 in PDAC cells using KRT19 as an epithelial tumour marker (Figure 2a).

**Figure 3:**
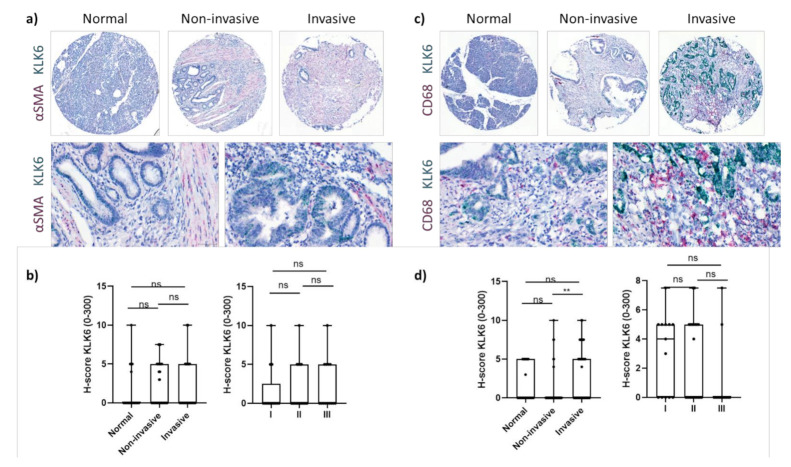
KLK6 mRNA expression in PDAC tissues. (**a**) Representative images of dual in situ hybridisation of KLK6 (green) and αSMA (red) in normal pancreas and PDAC tissues. (**b**) KLK6 was expressed by stromal cells at very low levels and did not correlate with (non)invasive tumour areas and tumour stage. (**c**) Representative images of dual in situ hybridisation of KLK6 (green) and CD68 (red) in normal pancreas and PDAC tissues. (**d**) KLK6 was expressed by CD68+ cells at very low levels and correlated with invasive tumour areas compared to the noninvasive counterparts. Asterisks indicate statistical significance (** *p* ≤ 0.01; ns, not significant).

KLK6 was highly expressed by PDAC cells independent of tumour stage (Figure 2b). Upon image quantification using QuPath, we found that the KLK6 mRNA levels were significantly upregulated in PDAC compared to normal pancreas tissues, with invasive tumour areas having significantly higher mRNA levels compared to noninvasive tumour areas (Figure 2c,d). In agreement with the APGI PDAC dataset, Kaplan–Meier analysis of our PDAC TME dataset also indicated that high KLK6 expression levels were significantly associated with shorter overall and disease-free survival than low expression levels (Figure 2e). In search for other KLK6-expressing cell types, we conducted a correlation analysis using the TCGA PDAC dataset and found that KLK6 correlated with tumour-associated genes present in the stroma and immune cells (Figure S1b,c). We then performed dual in situ hybridisation with αSMA, indicative of stromal cells, specifically cancer-associated fibroblasts, and CD68, representing macrophages. Both stromal cells and CD68+ cells expressed KLK6 at a very low level compared to PDAC cells (Figure 2d and Figure 3a,c). While KLK6-expressing stromal cells were not correlated with (non)invasive tumour areas and tumour stage (Figure 3b), KLK6-expressing CD68+ cells were present in invasive tumour areas but not in noninvasive tumour areas and did not correlate with tumour stage (Figure 3d).

**Figure 4:**
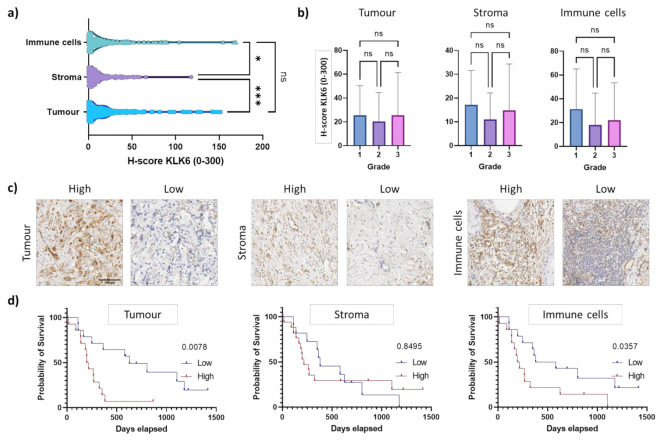
KLK6 protein expression in PDAC tissues. (**a**) KLK6 protein levels were significantly higher in the tumour and immune cells compared to the stroma. (**b**) KLK6 expression in tumour, stroma and immune cells did not correlate with tumour grade. (**c**) Representative images of the high and low KLK6 staining intensity in the tumour, stroma and immune cell areas. (**d**) Kaplan–Meier analysis indicated that high KLK6 expression levels in the tumour (*p* = 0.0078) and immune cells (*p* = 0.0357) were significantly associated with shorter survival than low expression levels (individuals from 2005 onwards). Scale bar, 100 µm. Asterisks indicate statistical significance (* *p* ≤ 0.05 and *** *p* ≤ 0.001; ns, not significant).

To determine the KLK6 protein expression levels, we performed immunohistochemical analysis using our own KLK6 antibody [24]. The H-score for the immunohistochemical analysis (mean of 23) was much lower compared to the dual in situ hybridisation (up to 300), suggesting a low protein expression (Figure 2d and Figure 4a). This is in line with an earlier in silico analysis demonstrating that the KLK6 mRNA is expressed at a high density in pancreatic cancer, whereas the KLK6 protein was undetectable in the pancreas using a highly sensitive immunofluorometric assay [10]. Upon immunohistochemical analysis using a different antibody compared to our study, the KLK6 protein was moderately expressed in the Langerhans’ islets and in PDAC tissues [7].

Cytoplasmic immunostaining was observed in epithelial tumour cells and in the surrounding stroma and immune cells, independent of tumour grade (Figure 4a–c). This is similar to the KLK6 staining pattern observed in ovarian cancer tissues [24]. Strikingly, high KLK6 protein levels in the tumour and immune cells were significantly associated with shorter survival compared to low protein levels, while there was no association of stromal KLK6 protein levels with survival (Figure 4d). This is in line with our dual in situ hybridisation analysis. Overall, our mRNA and protein expression data indicate that high KLK6 levels in tumour and immune cells are significantly linked to poor prognosis, while KLK6 levels in the stroma had no prognostic impact.

### 3.3. Effect of the APPI-4M KLK6 Inhibitor on Cell Functions and Tumour Spheroids

To investigate the tumour-biological role of KLK6 in PDAC, we performed functional assays and hydrogel-based tumour spheroids and tested the effect of our KLK6 inhibitor on mRNA expression, metabolic activity, cell proliferation, migration and protease secretion. We first assessed the mRNA expression of KLK6, and the other members of the PDAC-specific KLK cluster, in widely used human PDAC cell lines and colon cancer cells as KLK-expressing controls [10,25] (Figure S3). Then, we used the KLK6 antibody from the immunohistochemical analysis to confirm the protein expression in human PDAC cell lines, with Capan2 and MiaPaCa2 cells showing the highest KLK6 levels in whole-cell lysates (Figure S4). Next, we determined the effect of KLK6 inhibition on tumour cell proliferation and migration and treated a panel of PDAC cell lines with the same APPI-4M concentration (100 nM), which reduced the migratory behaviour of breast cancer cells in our earlier study [18]. However, cell proliferation and migration were largely not affected by KLK6 inhibition, with Capan2 cells showing an increased migratory ability and BxPC-3, MiaPaCa2 and Panc1 cells showing increased proliferative abilities (Figure S5a,b).

**Figure 5:**
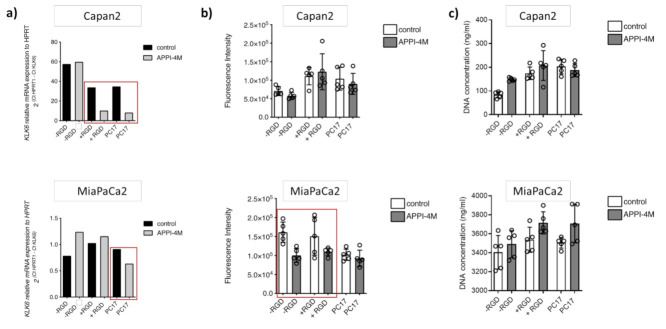
Effect of the APPI-4M KLK6 inhibitor on tumour spheroids. (**a**) KLK6 inhibition reduced the mRNA expression of Capan2 and MiaPaCa2 cells grown in PC17 and RGD-functionalised hydrogels (red boxes). (**b**) KLK6 inhibition reduced the metabolic activity (represented as fluorescence intensity) of MiaPaCa2 cells grown in RGD-functionalised hydrogels and hydrogels without RGD motif (red box). (**c**) KLK6 inhibition slightly increased cell proliferation (represented as DNA concentration) within all hydrogels tested.

To establish tumour spheroids, we set up 3D cultures with Capan2 and MiaPaCa2 cells grown embedded in PEG-based hydrogels of varying composition (without RGD motif, RGD-functionalised, PC17) for 14 days and treated them with the KLK6 inhibitor. Upon KLK6 inhibition, mRNA expression was reduced in both Capan2 and MiaPaCa2 cells grown in PC17 hydrogels, while Capan2 cells also had reduced mRNA levels in RGD-functionalised hydrogels (Figure 5a).

While the metabolic activity, indicative of cell viability, of Capan2 cells was not affected by the KLK6 inhibitor, MiaPaCa2 cells had a reduced metabolic activity in hydrogels independent of RGD functionalisation (Figure 5b). Proliferation in the 3D cell cultures was slightly increased upon KLK6 inhibition (Figure 5c).

**Figure 6:**
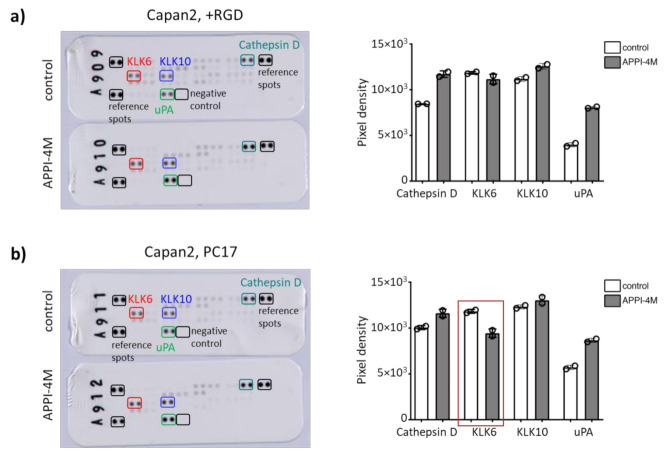
Effect of the APPI-4M KLK6 inhibitor on protease secretion of tumour spheroids. (**a**) KLK6 inhibition increased the secretion of cathepsin D and uPA in the conditioned medium of Capan2 cells grown in RGD-functionalised hydrogels. (**b**) Treatment with the KLK6 inhibitor reduced the secretion of KLK6 in the conditioned medium of Capan2 cells grown in PC17 hydrogels (red box), leading to an increased secretion of cathepsin D and uPA.

Of note, there was a trend that KLK6 inhibition reduced the metabolic activity of the control tumour spheroids using KLK6-expressing ovarian cancer cells (Supplementary Figure S6). Our hydrogels contained a proteolytic-degradable motif, and we wondered whether the mechanical properties, or stiffness, can be modified by KLK6 inhibition. Young’s modulus slightly declined in all hydrogel compositions tested upon KLK6 inhibition (Figure S7). To determine the changes in protease secretion, we assayed the conditioned medium from Capan2 cells grown in RGD-functionalised and PC17 hydrogels, which showed a reduced mRNA expression when treated with the KLK6 inhibitor. We found less KLK6 in the cell-conditioned medium from the PC17 hydrogel cultures upon KLK6 inhibition, while the secretion of cathepsin D and uPA was increased (Figure 6a,b). These proteases are part of proteolytic networks, which are dysregulated in cancer and other diseases like Parkinson’s disease [26,27]. Our findings indicate that the treatment of tumour spheroids with a proteolysis-resistant KLK6 inhibitor modified their mRNA expression, metabolic activity and secretion of KLK6 and other tumour-associated proteases.

## 4. Discussion

The tissue-specific expression of KLKs is modified in line with changing cell–cell and cell–matrix interactions during disease progression. KLKs are regulatory proteases and degrade numerous ECM proteins and other TME components, thereby activating proteolytic and signalling networks [7,8,9]. The pancreatic TME represents a rich substrate repertoire containing ECM proteins, cytokines, growth factors and a variety of other molecules, for example cell adhesion molecules. It has been reported that KLK6 and KLK7 cleave E-cadherin to promote tumour cell functions, including cell proliferation, migration and invasion [11,28].

The significant association of KLK expression levels and poor prognosis suggests that their inhibition may be a therapeutic strategy for precision medicine. Although KLK10 showed higher mRNA levels compared to KLK6 in the TCGA PDAC dataset, we focused on KLK6, as we previously reported a newly developed monospecific polyclonal antibody suitable for immunohistochemical analysis [24] and a potent KLK6-targeted inhibitor that is resistant to proteolysis [18]. Of note, the TCGA PDAC dataset contains a very low number of normal pancreas tissues [22]. Only one other study reported the upregulation and coexpression of KLK6 and KLK10 in PDAC and their association with poor prognosis [7]. While the tumour-biological role of KLK10 in PDAC was assessed by gene silencing and a cell migration assay, the role of KLK6 was not further investigated (Table S2). Here, we corroborate that KLK6 is linked to poor prognosis and associated with the TME of PDAC.

Our mRNA and protein expression analyses using two different PDAC patient cohorts demonstrate that high KLK6 levels in tumour and immune cells are significantly linked to poor prognosis. KLK6 expression in the stroma had no prognostic impact. In line with another study [24], we found KLK6 expressed in tumour-adjacent cells. By dividing our patient cohort into low and high expressers, we found a significant correlation with high KLK6 protein levels in KRT19+ and CD68+ cells with shorter survival compared to low protein levels, while there was no association of KLK6 protein levels with survival in αSMA+ cells. The localised KLK6 expression in tumour cells (identified by KRT19) and macrophages (represented by CD68) in the invasive areas underscores the potential for KLK6-targeted inhibitors that interfere with tumour-biological events in a context-dependent manner.

Additionally, KLK6 expression seems to correlate with tumour differentiation. Well-differentiated ducts harbour very low levels of KLK6, while undifferentiated tumours, with nuclear atypia and single-cell invasion, tend to have a higher KLK6 expression. In ovarian cancer, high KLK6 levels were detected in the stromal cells in poorly differentiated tumours (nuclear grade G3 versus G1/2) [24]. In PDAC, an increased KLK7 expression was associated with lower-grade moderate or well-differentiated tumours [8]. These expression patterns are context-dependent and may be further explored in future studies. In terms of the immune cell population, high KLK6-expressing immune cells are more likely to be macrophages, as indicated by our dual KLK6/CD68 in situ hybridisation. Low KLK6-expressing immune cells potentially include leucocytes or lymphocytes according to the observed morphology. In our future studies, we will perform a dual KLK6/CD45 or KLK6/CD3 in situ hybridisation to confirm this aspect.

KLKs are expressed in other gastrointestinal cancers, including colon cancer, and are hormonally regulated [25]. Previous studies have shown that KLK6 is regulated by oestrogen [10]. KLK6 is found in the Langerhans’ islets, which contain hormone-producing cells. Pancreatic neuroendocrine tumours, a less common subtype of pancreatic cancer, arise from the hormone-producing cells of the Langerhans’ islets. Our future studies may be aimed at investigating the relationship between KLK6 and pancreatic neuroendocrine tumours.

KLK6 has been associated with other cancer types, for example melanoma, glioblastoma multiforme, non-small-cell lung cancer and oral squamous cell carcinoma, the central nervous system and inflammatory conditions, such as in skin and joint diseases, and signals through G protein-coupled proteinase-activated receptors (PARs) [15,29,30,31,32,33]. The inflammatory microenvironment of tumours and other diseases interlinks KLKs and other tumour-associated proteases with PAR-mediated signalling events, which are activated by proteolytic cleavage [34].

In human melanoma tissues, KLK6 expression was found in the stroma area in the inflammatory TME and was associated with the activation of PAR1, triggering intracellular calcium flux and tumour cell invasion [29]. The KLK6/PAR1 axis was linked to glioblastoma multiforme and injuries of the central nervous system [15,30]. KLK6 regulated the proliferation and apoptosis of non-small-cell lung cancer cells via cleavage and activation of PAR2, which in turn activated the ligand-dependent epidermal growth factor receptor (EGFR) pathway [31]. KLK6 contributed to the malignant transformation of oral epithelial cells via the activation of PAR2, but not PAR1, and ERK signalling [32]. In inflamed tissues from psoriatic lesions, KLK6 was found coexpressed with KLK7-10, KLK13 and PAR1. Thus, in a recent study, the FDA-approved PAR1 antagonist vorapaxar was tested as a treatment of inflammatory skin diseases [33]. High KLK6 expression levels were linked to inflammatory skin diseases, such as severe psoriasiform dermatitis. In these patients, KLK6 may exert its proinflammatory effects via proteolytic cleavage of PAR1. Treatment of patient-derived psoriatic skin tissues with the PAR1 antagonist caused a downregulation of psoriasis-associated inflammatory markers [33]. In our study, we identified elevated expression of KLK6 in PDAC, and targeting KLK6/PAR1 signalling may be a promising therapeutic strategy to slow down tumour growth. Our preclinical tumour spheroid model may be applied to test the PAR1 antagonist and to decipher the role of the KLK6/PAR1 axis, or other PARs, in PDAC.

The role of KLK6 in pancreatic cancer might be further explored in vivo by using for example an orthotopic xenograft approach, whereby tumour spheroids established from KLK6-expressing and KLK6-deficient PDAC cells are positioned adjacent to the pancreas [4]. The use of immunodeficient animals will allow the inclusion of patient-derived stromal and immune cells, for example cancer-associated fibroblasts and macrophages.

Preclinical models that closely recapitulate disease biology improve our understanding of the cellular responses at the molecular level and reveal novel combinations of therapeutic agents [35]. Combining preclinical models with machine learning approaches that utilise large databases and experimental data may identify tumour cell responses to therapeutic agents, for example by linking treatment responses to the mutational status of KLK6 [36]. Strategies for developing KLK-targeted therapeutic agents include the inhibition of their proteolytic activity, KLK-activated prodrugs, delivery of cytotoxic genes under the control of KLKs promoter/enhancer elements or KLK-based immunotherapies [5]. Here, we tested a KLK6 inhibitor that is resistant to proteolysis and its therapeutic potential for the treatment of PDAC.

The APPI-4M KLK6 inhibitor was designed by a combinatorial screening approach, that included a flow cytometry-based screening of a yeast surface-displayed mutant library, to specifically target KLK6. APPI-4M is a human amyloid precursor protein inhibitor that contains the Kunitz-type protease inhibitor domain with high binding affinity to KLK6. As reported, APPI-4M significantly reduced the migratory and invasive behaviour of the epithelial breast cancer BT-20 cell line over 24 and 36 h, respectively [18]. However, BT-20 cells originate from a triple-negative breast tumour and do not account for the hormone-dependent regulation of KLKs mentioned earlier and their expression in hormone-dependent malignancies, such as hormone-receptor-positive breast cancer, accounting for the majority of breast tumours, as well as prostate cancer or ovarian cancer [12]. While high levels of secreted KLK6 were found in pancreatic cancer MiaPaCa2 cells, secretion of KLK6 was not detected in the BT-20 cells [12]. This is contrary to an earlier study, reporting the presence of KLK6 in the conditioned medium of BT-20 cells [37]. In our study, we tested the effect of the same APPI-4M concentration (100 nM) on the cell proliferation and migration of a panel of PDAC cell lines.

Following KLK6 inhibition, we observed an increased proliferation and migration of KLK6-expressing MiaPaCa2 and Capan2 cells, respectively. Capan2 cells had the highest doubling time of all PDAC cells tested [38]. Thus, it was not surprising that the migratory behaviour of Capan2 cells rather than their proliferative behaviour changed upon KLK6 inhibition. Upon adhesion to collagen type-I and treatment with our KLK6 inhibitor, the proliferation of KLK6-deficient BxPC3 and Panc1 cells was increased. This points towards compensatory responses of other KLKs and proteolytic factors, which may be stimulated by collagen. Collagen is involved in the epithelial-to-mesenchymal transition (EMT) in fibrotic tissues, malignant transformation and during tumour metastasis [5,29,32]. KLK6 has also been reported to regulate factors and cell functions associated with EMT events [39]. Overall, we observed a varying proliferative and migratory response to KLK6 inhibition in our PDAC cells, which may be linked to their different phenotypes. While BxPC3 cells are epithelial, MiaPaCa2 have a mesenchymal-like phenotype. PANC-1 cells present an intermediate profile, displaying an epithelial phenotype with some mesenchymal-like aspects [40]. We will need to conduct further studies to elucidate the precise role of KLK6, and other KLKs, and the inhibition of protease activity in PDAC cells, including determining the effect of KLK6 inhibition on their invasive abilities.

Following KLK6 inhibition, KLK6 mRNA expression; metabolic activity, indicative of cell viability; and secretion of KLK6 were reduced in our tumour spheroids, while the secretion of cathepsin D and uPA was increased. The metabolic activity and proliferation of tumour spheroids formed by MiaPaCa2 cells was generally higher compared to Capan2 cells. KLK6 inhibition greatly reduced the mRNA expression and metabolic activity of MiaPaCa2 spheroids, while Capan2 spheroids had a consistently reduced mRNA expression. However, proliferation in the 3D cell cultures was slightly increased upon KLK6 inhibition, which is in line with our cell monolayer data, in particular for the MiaPaCa2 cells. We performed the 3D cell culture experiments using hydrogels of varying compositions (without RGD motif, RGD-functionalised, PC17) and will need to determine in our future studies whether the presence of the RGD motif impacts the cell’s responses to KLK6 inhibition. We detected consistent results in terms of the reduced KLK6 and increased cathepsin D and uPA secretion, suggesting that APPI-4M specifically inhibits KLK6, which caused a compensatory response of these serine and aspartic lysosomal proteases. KLK6 interacts with other secreted factors as part of proteolytic networks, which are dysregulated in cancer and other diseases [5,26,27,41]. In a proteomic analysis, cathepsin D and its family member cathepsin B were identified as interacting partners of the anterior gradient 2 (AGR2) protein in pancreatic cancer cells and involved cell dissemination. Interestingly, the proteolytic inactive proform of cathepsin D was detected in the-cell conditioned medium and has also been reported in other metastatic cancers [42].

The interplay of KLKs with tumour-associated proteases such as uPA has been implicated in the regulation of cell migration and dissemination [26]. The serine protease uPA catalyses the conversion of plasminogen to plasmin, and its receptor uPAR has been studied in pancreatic cancer cells. Shed uPAR was detected in conditioned medium of KLK7-expressing cells, which also had a decreased cell adhesion to vitronectin. This was not surprising, as uPAR mediated adhesion to vitronectin [43]. The APPI-4M KLK6 inhibitor was reported to act like a functional inhibitor blocking tumour cell functions, such as cell migration [18]. The observed effect on the mRNA expression has not been investigated before, and thus, it also acts as a pharmacological knockdown. Our findings confirm the KLK6-specific effects as APPI-4M reduced the KLK6 secretion of pancreatic cancer cells, while KLK10 secretion was not affected. Reduced KLK6 secretion counter-regulates cathepsin D and uPA. Cathepsin D and both uPA and uPAR have been found highly expressed in PDAC and play a role in cell dissemination, which ultimately leads to tumour progression [44]. Although KLK6 expression was not correlated with tumour stage in our patient cohort, our findings suggest that a KLK6-targeted therapeutic intervention might be beneficial at the earlier tumour stage prior to the development of an invasive tumour area. This may prevent the compensatory response of other proteolytic factors. In future studies, we will investigate the interrelationship within this proteolytic network by using for example a CRISPR/Cas9 KLK6 knockout or a double knockout approach. While we focused on studying the role of KLK6 in PDAC, the role of the other coexpressed KLKs, in particular KLK10, needs to be further investigated, for example by using a KLK6 and KLK10 double knockout.

In cell-conditioned medium of cortical neurons, KLK6 activated matrix metalloproteinase 2 (MMP2) and a disintegrin and metalloproteinase with thrombospondin motif 19 (ADAMTS19), all members of the proteolytic and regulatory network in neurological disorders, triggering α-synuclein processing [45]. Our findings are in line with these reports and point towards a correlation between KLK levels and other proteolytic factors present within the tumour and its extracellular and cellular microenvironment. Interactions between different KLKs and their proteolytic network are highly complex and context dependent. By using specific antibodies for analysis and specific inhibitors targeting single KLKs, these interactions may be decoded or disrupted, revealing their individual functions and eventually reducing disease progression.

## 5. Conclusions

KLKs have important roles in the pancreatic TME and represent a therapeutic approach for inoperable tumours or an addition to standard-of-care therapy for PDAC. Because the majority of patients present with inoperable, locally advanced or metastatic disease, a KLK-targeted therapeutic intervention may have a positive impact on the survival of this patient population. KLK6-targeted inhibition reduced KLK6 mRNA expression, secretion and metabolic activity of tumour spheroids, which in part modified its tumour-biological role in cell dissemination. By applying our preclinical tumour spheroid model, we aim to test in our future studies targeted inhibitors or antagonists in combination treatment strategies with chemotherapeutics.

## Data Availability

The data presented in this study are available on request from the corresponding author.

## Supplementary Materials

The following are available online at https://www.mdpi.com/article/10.3390/cancers13163969/s1, Figure S1: KLK co-expression using the TCGA PDAC dataset, Figure S2: KLK co-expression using the KLK-CANMAP, Figure S3: KLK mRNA expression in human cancer cells, Figure S4: KLK6 protein expression in human pancreatic ductal adenocarcinoma cells, Figure S5: Effect of the APPI-4M KLK6 inhibitor on tumour cell proliferation and migration, Figure S6: Effect of the APPI-4M KLK6 inhibitor on tumour spheroids, Figure S7: Mechanical properties of cell-containing hydrogels, Table S1: Details of individuals diagnosed with pancreatic adenocarcinoma following the grading and staging of the World Health Organization criteria and specimen used to generate our tissue microarray, Table S2: Comparison of the study by Rückert et al and our findings.
