# Supplementary material for: Kallikrein-Related Peptidase 6 Is Associated with the Tumour Microenvironment of Pancreatic Ductal Adenocarcinoma
Source: Cancers (Basel). 2021 Aug 5;13(16):3969. doi: 10.3390/cancers13163969 (PMC8392253; doi:10.3390/cancers13163969)
Supplement: Supplementary file 1 [file cancers-13-03969-s001.zip › cancers-1267171-supplementary.pdf]

Kallikrein-Related Peptidase 6 Is Associated with the Tumour Microenvironment of Pancreatic Ductal Adenocarcinoma

Juliana B. Candido, Oscar Maiques, Melanie Boxberg, Verena Kast, Eleonora Peerani, Elena Tomás-Bort, Wilko Weichert, Amiram Sananes, Niv Papo, Viktor Magdolen, Victoria Sanz-Moreno and Daniela Loessner

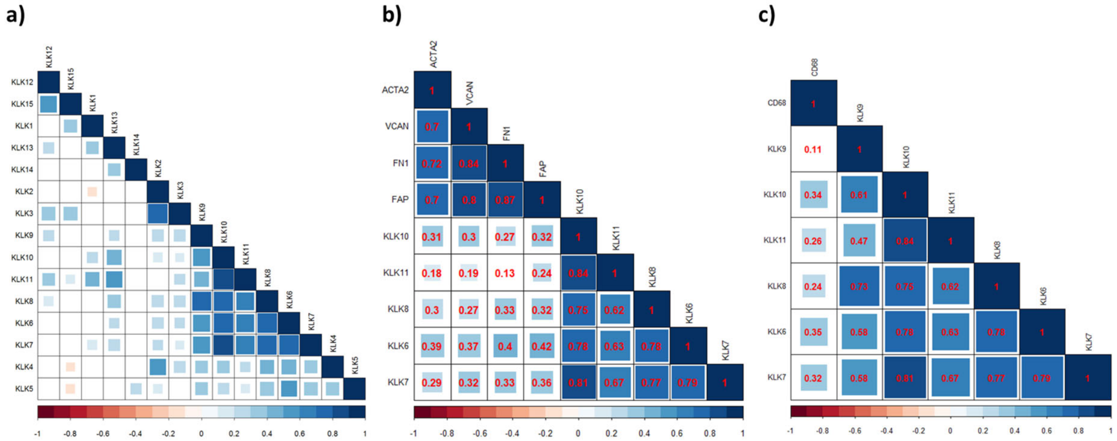

Figure S1. KLK co-expression using the TCGA PDAC dataset. (**a**) Correlation analysis of the 15 members of the KLK family indicating the PDAC-specific KLK cluster that includes KLK6, KLK7, KLK8, KLK10 and KLK11. (**b**) Out of the PDAC-specific KLK cluster, KLK6 correlated with tumour-associated genes present in the stroma. (**c**) Out of the PDAC-specific KLK cluster, KLK6 correlated with the tumour-associated macrophage marker CD68.

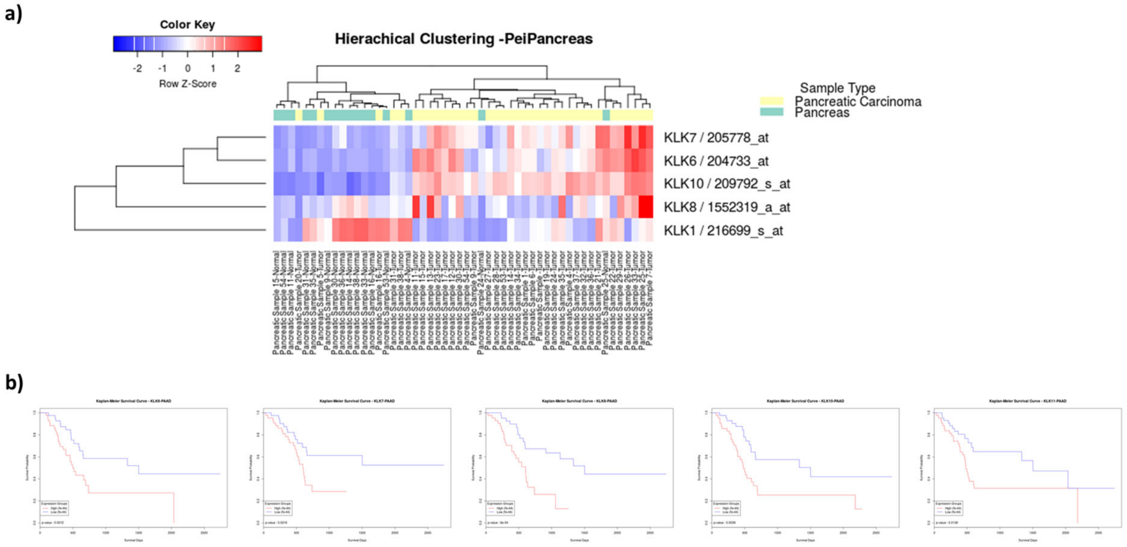

Figure S2. KLK co-expression using the KLK-CANMAP. (**a**) KLK6, KLK7, KLK8 and KLK10 were identified as highly upregulated in PDAC compared to normal pancreas tissues, which showed an upregulation of KLK1. (**b**) Kaplan-Meier analysis indicated that high KLK6 (p = 0.0072), KLK7 (p =

0.0216), KLK8 (p = 0.0008), KLK10 (p = 0.0039) and KLK11 (p = 0.0138) expression levels were significantly associated with a shorter 5-year survival than low expression levels.

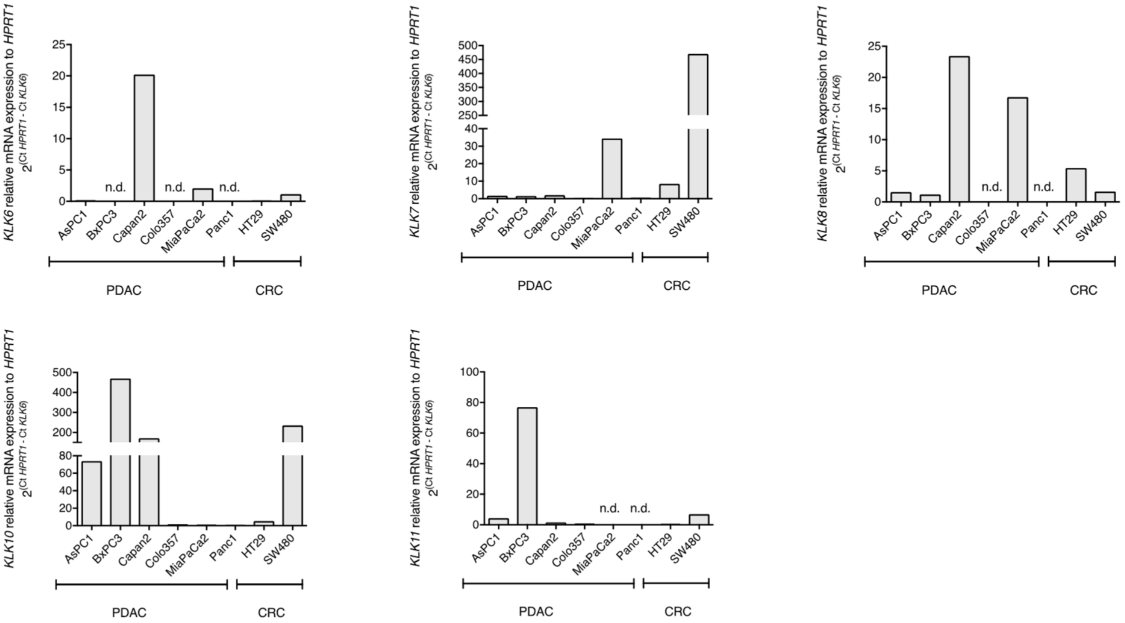

Figure S3. KLK mRNA expression in human cancer cells. Gene expression analysis of KLK6, KLK7, KLK8, KLK10 and KLK11 in human pancreatic ductal adenocarcinoma (PDAC) and colorectal cancer (CRC) cells. n.d., not determined.

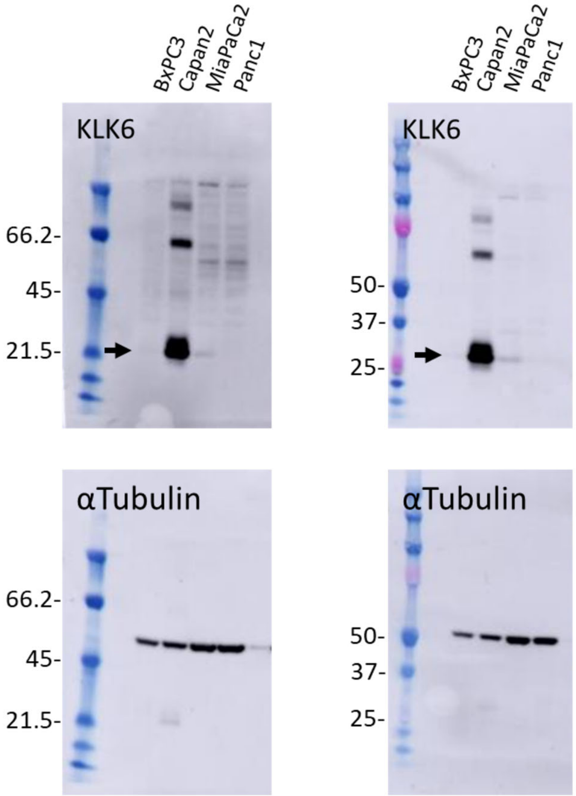

Figure S4. KLK6 protein expression in human pancreatic ductal adenocarcinoma cells. Protein expression analysis of KLK6 (arrows; 26.8 kDa) in whole-cell lysates using two different molecular weight ladders.

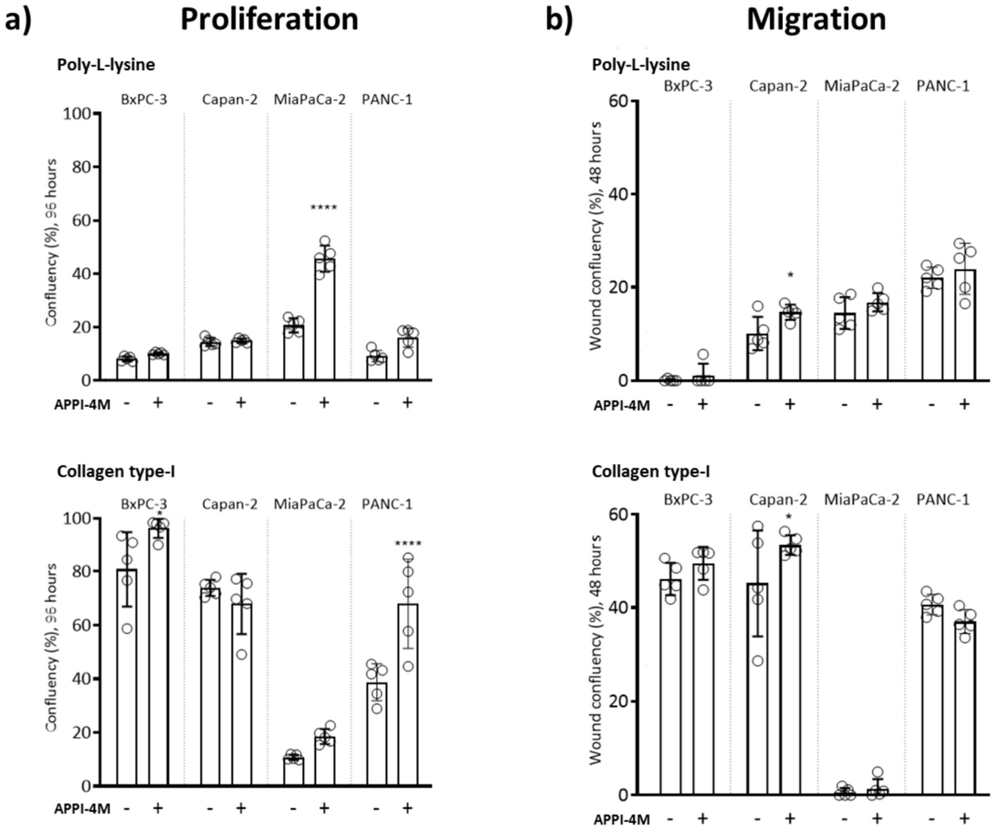

Figure S5. Effect of the APPI-4M KLK6 inhibitor on tumour cell proliferation and migration. (**a**) Incucyte proliferation analysis of human pancreatic ductal adenocarcinoma cells using poly-L-lysine and collagen type-I-coated plates after 96 hours ± KLK6 inhibition. (**b**) Incucyte migration analysis of human pancreatic ductal adenocarcinoma cells using poly-L-lysine and collagen type-I-coated plates after 48 hours ± KLK6 inhibition.

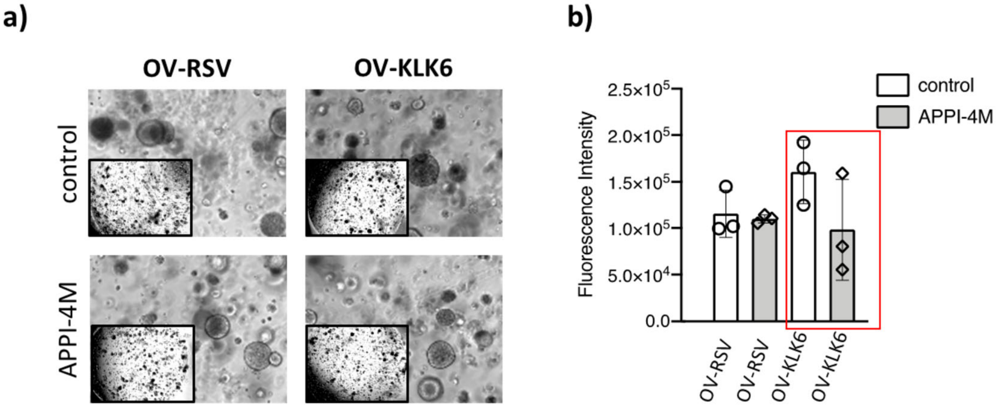

Figure S6. Effect of the APPI-4M KLK6 inhibitor on tumour spheroids. (**a**) Representative brightfield micrographs of ovarian cancer cells grown in polyethylene glycol-based hydrogels over 14 days ± KLK6 inhibition; magnification 20×, insert 4×. (**b**) KLK6 inhibition reduced the metabolic activity of KLK6-expressing tumour spheroids (OV-KLK6; red box) compared to vector controls (OV-RSV).

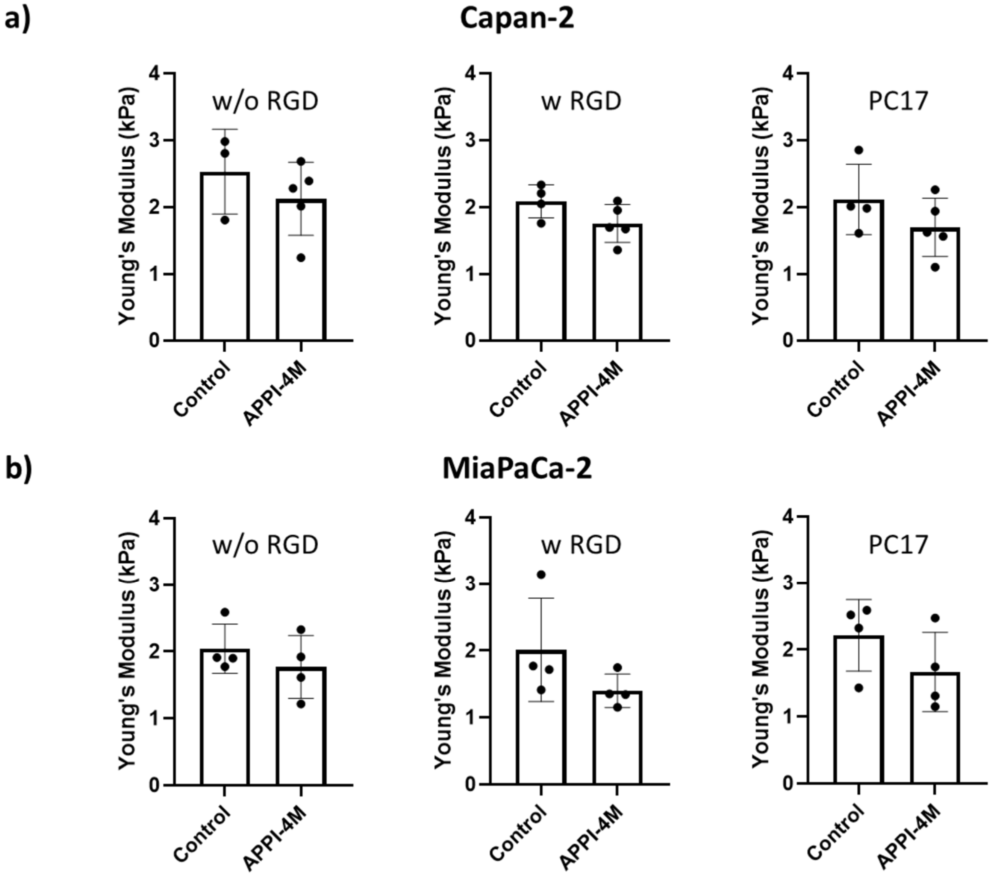

Figure S7. Mechanical properties of cell-containing hydrogels. (**a**) Young's modulus of Capan2-containing hydrogels of varying composition (without RGD motif, RGD-functionalised, PC17) ± KLK6 inhibition. (**b**) Young's modulus of MiaPaCa2-containing hydrogels of varying composition (without RGD motif, RGD-functionalised, PC17) ± KLK6 inhibition.

Table S1. Details of individuals diagnosed with pancreatic adenocarcinoma following the grading and staging of the World Health Organization criteria and specimen used to generate our tissue microarray. T, tumour; N, nodes.

Patient	Gender	Age	Size (mm)	T stage	N Stage	Stage
1	Female	77	30	pT2	pN0	IB
2	Male	68	22	pT2	pN0	IB
3	Male	41	30	pT2	pN1	IIB
4	Male	56	23	pT2	pN1	IIB
5	Male	54	19	pT1c	pN1	IIB
6	Male	62	23	pT2	pN0	IB
7	Female	74	39	pT2	pN1	IIB
8	Female	68	20	pT1c	pN2	III
9	Female	75	34	pT4	pN1	III
10	Male	83	11.5	pT1c	pN0	IB
11	Male	70		cT4		III
12	Female	66	28	pT2	pN1	IIB
13	Female	74	27	pT2	pN1	IIB
14	Male	68	30	pT2	pN1	IIB
15	Female	62	25	pT2	pN1	IIB
16	Male	75	28	pT2	pN0	IB
17	Female	74	29	pT2	pN0	IB
18	Female	63	16	pT1c	pN0	IA
19	Male	56	26	pT2	pN1	IIB
20	Female	70	26	pT2	pN1	IIB

21	Female	70		cT4	pN1	III
22	Male	75	39	pT4	pN1	III
23	Male	64	11	pT1a	pN0	I
24	Male	81	25	pT2	pN2	III
25	Female	76	17	pT1c	pN0	IA
26	Male	69	55	pT3	pN2	III
27	Female	62	24	pT2	pN0	IB
28	Male	60	26	pT2	pN1	IIB
29	Female	81	19	pT1c	pN0	IA
30	Male	82	20	pT1c	pN1	IIB
31	Male	64	68	pT3	pN1	IIB
32	Male	62	27	pT2	pN1	IIB
33	Male	63	32	pT2	pN1	IIB
34	Male	41	28	pT2	pN1	IIB
35	Male	81	25	pT3	pN2	IIIB
36	Male	74	24	pT3b	pN0	IIB
37	Male	74	28	pT3	pN0	IB
38	Male	77	33	pT2	pN2	III
39	Female	73	55	pT3	pN1	IIB
40	Female	70	27	pT2	pN2	III
41	Male	81		pT4		III

Table S2. Comparison of the study by Rückert et al and our findings. APGI, Australian Pancreatic Cancer Genome Initiative; Col-I, collagen type-I; KLK, kallikrein-related peptidase; PDAC, pancreatic ductal adenocarcinoma; TCGA, The Cancer Genome Atlas.

	**Rückert et al. 2008**	**Our study**
investigated the KLK network in PDAC using publicly available datasets	high-density DNA microarray *n* = 19	TCGA dataset *n* = 178, APGI dataset *n* = 456
investigated own PDAC patient cohort	*n* = 54	*n* = 262 (German cohort), *n* = 41 (Spanish cohort)
co-expression	KLK6 and KLK10	KLK6, KLK7, KLK8, KLK10 and KLK11
associated with shorter survival	co-expression of KLK6 and KLK10	high KLK6 levels in tumour and CD68+ cells, high KLK7 level, high KLK10 level, high KLK11 level
KLK6 mRNA expression	cell lines	tissues and cell lines, in tumour, stromal and CD68+ cells
KLK6 protein expression	tissues and cell lines	tissues and cell lines, in tumour, stromal and immune cells
KLK6 serum expression	patient-derived samples	
cell proliferation		3 out of 4 cell lines increased on Col-I
cell migration	reduced in KLK10 knockout cells	3 out of 4 cell lines increased on Col-I
tumour spheroid formation		hydrogel-based assay
investigated KLK6 interaction partners	in silico analysis	human protease array
tested KLK6 inhibition		used the proteolysis-resistant KLK6 inhibitor APPI-4M
effects of KLK6 inhibition		reduced mRNA expression, reduced cell metabolic activity, reduced KLK6 secretion, increased secretion of urokinase plasminogen activator and cathepsin D
